# Effect of High-Fat Diet on Peripheral Neuropathy in C57BL/6 Mice

**DOI:** 10.1155/2014/305205

**Published:** 2014-10-27

**Authors:** Lingling Xu, Dou Tang, Meiping Guan, Cuihua Xie, Yaoming Xue

**Affiliations:** Department of Endocrinology, Nanfang Hospital, Southern Medical University, Guangzhou 510515, China

## Abstract

*Objective*. Dyslipidemia may contribute to the development of peripheral neuropathy, even in prediabetics; however, few studies have evaluated vascular dysfunction and oxidative stress in patients with peripheral neuropathy. *Methods*. Using high-fat diet- (HFD-) induced prediabetic C57BL/6 mice, we assessed motor and sensory nerve conduction velocity (NCV) using a BIOPAC System and thermal algesia with a Plantar Test (Hargreaves' method) Analgesia Meter. Intraepidermal nerve fiber density and mean dendrite length were tested following standard protocols. Vascular endothelial growth factor-A (VEGF-A) and 12/15-lipoxygenase (12/15-LOX) were evaluated by immunohistochemistry and Western blot, respectively. *Results*. HFD-fed mice showed deficits in motor and sensory NCV, thermal hyperalgesia, reduced mean dendrite length, and VEGF-A expression in the plantar skin and increased 12/15-LOX in the sciatic nerve (*P* < 0.05 compared with controls). *Conclusion*. HFD may cause large myelinated nerve and small sensory nerve fiber damage, thus leading to neuropathy. The mean dendrite length may be a more sensitive marker for early detection of peripheral neuropathy. Reduced blood supply to the nerves and increased oxidative stress may contribute to the development and severity of peripheral neuropathy.

## 1. Introduction

Diabetic peripheral neuropathy (DPN) is a common cause of morbidity and is a leading cause of foot amputation, resulting in a huge economic burden [1]. Due to the complex and poorly understood pathogenesis of DPN [2], treatment is very challenging. It has been shown that hyperglycemia plays an important role in the development of DPN, at least partially through systemic and neuronal oxidative stress [3, 4].

A previous study showed that obese patients with normal glucose levels have symptoms of neuropathy, suggesting an association between obesity and peripheral neuropathy [5]. Furthermore, a growing body of evidence is emerging with respect to the development of peripheral neuropathy in the prediabetic stage in clinical [6, 7] and experimental studies [8, 9].

The prediabetic stage, especially in the context of metabolic syndrome (MS) [7, 10], is a leading cause of peripheral neuropathy, accounting for approximately 35.5% of undiagnosed cases [6]. Accumulating evidence suggests that dyslipidemia may contribute to the development of peripheral neuropathy. The European Outpatients with Type 1 Diabetes (EURODIAB) study established a significant association between fasting lipids and the development of neuropathy, even in prediabetic patients [11]. In 2009, Wiggin and colleagues [12] reported that elevated triglyceride levels are associated with a more rapid disease course in patients with neuropathy. Additionally, in a cross-sectional study with 548 type 2 diabetic patients, it has been shown that those with MS were twice as likely to develop DPN, for which dyslipidemia is the major driver [13].

It is known that a high-fat diet (HFD) can affect glucose metabolism, and dietary fat is associated with the conversion of impaired glucose tolerance (IGT) to type 2 diabetes [14]. HFD-fed mice have been used for studying the pathogenesis of neuropathic changes in IGT, obesity, and dyslipidemia. It has been documented that HFD-fed C57BL/6 mice develop peripheral neuropathy, even with relatively normal glucose levels [8, 9].

In our previous pilot study [15], we showed that HFD causes peripheral neuropathy in C57BL/6 mice; collectively, the decreased thermal pain threshold, reduced nerve conduction velocity (NCV), and increased T-SOD activity and MDA content were suggestive of an oxidant/antioxidant balance disorder. In the current study we increased the sample size and investigated the underlying mechanism between a HFD and the development of peripheral neuropathy. In previous clinical studies [7, 16], the mean dendrite length was used as a marker for early neuropathy rather than the intraepidermal nerve fiber density. Furthermore, it remains unclear whether or not vascular endothelial growth factor-A (VEGF-A), which promotes vascular endothelial mitosis and new blood vessel formation in mouse plantar skin [17], plays a role during the development of DPN. A recent study suggested that 12/15-lipoxygenase (12/15-LOX) inhibition counteracts diabetes related MAPK phosphorylation in mouse and cell culture models of diabetic neuropathy and implies that 12/15-LOX inhibitors may be an effective treatment for DPN [18]. In the current study, using HFD-induced prediabetic neuropathy mice, we determined the potential mechanism involving these two major pathways and further investigated their possible correlations with peripheral neuropathy in prediabetic mice.

## 2. Materials and Methods

### 2.1. Research Design and Methods

The current study was approved by the Ethics Committee of Nanfang Hospital, affliated to Southern Medical University (Guangzhou, China). All animal experiments were conducted according to the Principles of Laboratory Animal Care (National Institutes of Health (NIH) publication number 85-23, revised 1985) and Pennington Biomedical Research Center protocols. The protocols were in accordance with the institutional guidelines for the care and use of laboratory animals at Southern Medical University (Guangzhou, China).

### 2.2. Reagents

Unless otherwise stated, all chemicals were of reagent-grade quality from Sigma-Aldrich (St. Louis, MO, USA). Rabbit polyclonal anti-protein gene product 9.5 (ubiquitin COOH-terminal hydrolase) antibody was purchased from Chemicon International (Temecula, CA, USA). Rabbit polyclonal anti-VEGF-A and rabbit polyclonal (clone H-100) anti-12-lipoxygenase antibodies were obtained from Santa Cruz Biotechnology, Inc. (Santa Cruz, CA, USA).

### 2.3. Animals

Female C57BL/6 mice (3 weeks old) were purchased from The Jackson Laboratory (Bar Harbor, ME, USA). After 1 week of adaptation, animals were divided into two groups (15 mice/group) and provided a control (10 kcal% fat) or HFD (45 kcal% fat, D12451i; Research Diets, New Brunswick, NJ, USA). Diets were matched for protein, carbohydrate content, calories, and density. Five mice were housed in one cage in a temperature-controlled room under a 12-hour light/dark cycle with free access to food and water.

### 2.4. Intraperitoneal Glucose Tolerance Test (IPGTT)

An IPGTT was performed at a fixed time every other week after 6 weeks of dietary treatment. After 6 hours of fasting, one drop of tail blood was collected for glucose using a standard glucometer (One Touch Profile, Lifescan Inc., Milpitas, CA, USA, #6 strips). Then, glucose was injected intraperitoneally into conscious mice at a dosage of 1 g/kg body weight. Blood glucose values were obtained after 30, 60, and 120 min. Glucose tolerance was indicated as the area under the curve (AUC), which was calculated by the trapezoid rule from the glucose measurements at 0 (fasting), 30, 60, and 120 min.

### 2.5. Thermal Algesia and NCV

Hind-paw thermal latency was measured by determining changes in paw withdrawal latency (PWL) using a plantar algesia apparatus (model number 33, Analgesia Meter IITC, Life Science Instruments, Woodland Hills, CA, USA). NCVs were measured in compliance with protocols established by the AMDCC (http://www.diacomp.org). Mice were anesthetized with 100/10 mg/kg ketamine/xylazine by intraperitoneal injection, and body temperature was maintained at 32–34°C using a heating pad.

### 2.6. Tissue Harvest

Mice were euthanized with an overdose of sodium pentobarbital when HFD-fed mice first exhibited signs of impaired glucose tolerance (IGT). Blood samples were collected into Eppendorf tubes and centrifuged at 1500 rpm for 15 min at 4°C. Serum was collected and stored at −80°C until analysis. Sciatic nerves were rapidly dissected and frozen in liquid nitrogen for subsequent 12/15-LOX determination. Two skin biopsy samples were collected from the hind-paw plantar surfaces of each mouse.

### 2.7. Plasma Assays

Serum triglycerides (TG), total cholesterol (TC), high-density lipoprotein cholesterol (HDL-C), and low-density lipoprotein cholesterol (LDL-C) were measured using Wako kits (Wako Pure Chemical, Osaka, Japan) and a Beckman CX7 analyzer (Beckman Coulter, Brea, CA, USA).

### 2.8. Intraepidermal Nerve Fiber Density and Mean Dendrite Length

The first specimen was immediately postfixed in Zamboni's solution (2% paraformaldehyde (PFA) and 1% picric acid in 0.1 M phosphate-buffered saline (PBS)) overnight and then rinsed in 5%, 10%, and 20% sucrose in 50 mM sodium phosphate buffer. The samples were then snap-frozen in optimal cutting temperature (OCT) compound and stored at −80°C. Three longitudinal 45 um thick footpad sections from each mouse were cut on a Leica CM1950 cryostat (Leica Microsystems, Wetzlar, Germany). After quenching with 3% H_2_O_2_ and blocking with 5% goat serum, rabbit polyclonal anti-protein gene product 9.5 antibody was applied at a 1 : 1000 dilution. Secondary biotinylated goat anti-rabbit IgG antibody was applied at a 1 : 400 dilution, and staining was performed with the Vectastain Elite ABC kit (Vector Labs, Burlingame, CA, USA). Specific binding was visualized with a DAB substrate kit containing 3,3-diaminobenzidine (DAB; Sigma-Aldrich, MO, USA).

Intraepidermal nerve fibers were evaluated by three independent investigators in a blinded fashion under an Axioplan 2 microscope (Zeiss, Oberkochen, Germany) at 40x magnification, and the mean values were used for analysis. The width of epidermis was assessed using micrographs of stained sections taken at 5x magnification using Image software (NIH, Bethesda, MD, USA). An average of 2.8 ± 0.3 mm of the sample length was investigated to calculate intraepidermal nerve fibers per mm of epidermis and the mean dendrite length was reported in *μ*m.

### 2.9. VEGF-A Immunostaining

The second biopsy sample was fixed in 4% PFA in PBS for 12–24 h, dehydrated in a graded ethanol series and xylene, paraffin-embedded, and cut into 5 um sections. Three randomly chosen sections from each mouse were mounted. The sections were dewaxed, rehydrated, and microwave pretreated in 10 mM citrate buffer for 20 min at 750 W for antigen retrieval. After quenching with 3% H_2_O_2_ and blocking with 5% normal goat serum, rabbit polyclonal antibody to VEGF-A was applied at a 1 : 300 dilution. Secondary goat anti-rabbit IgG antibody was applied at a 1 : 400 dilution for approximately 1 h. A Vectastain Elite ABC kit (Vector Labs, Burlingame, CA, USA)was used for staining, as described above. Negative controls were sections that underwent the same treatment except that the primary antibody was omitted.

The protocol for VEGF-A assessment included a fixed light intensity, a condenser set between 2 and 3, and default Nikon color settings. Every image was evaluated using a standardized Leica program to quantify the amounts of stained and total areas (Leica QWin Standard V2.4). The basal, granular, and spinous layers of the epidermis were assessed with the keratin layer excluded. The positively stained area was divided by the total area to quantify the amount of staining as a percentage of the total area. All observations were performed on coded slides to reduce observer bias.

### 2.10. Western Blot Analysis of 12/15-LOX Protein Expression

To assess 12/15-LOX expression by Western blot analysis, sciatic nerve material (~10 mg) was placed on ice in 200 *μ*L of radioimmunoprecipitation assay (RIPA) buffer, as per protocol, and then homogenized on ice. The homogenates were sonicated (3 × 5 s) and centrifuged at 14000 ×g for 20 min. All of the aforementioned steps were performed at 4°C. The supernatant lysates (20 *μ*g of protein) were mixed with equal volumes of 2× sample-loading buffer containing 62.5 mmol/L Tris-HCl (pH 6.8), 2% SDS, 5% *β*-mercaptoethanol, 10% glycerol, and 0.025% bromophenol blue and separated on 10% SDS-polyacrylamide gels by electrophoresis at 15 mA constant current for stacking and at 25 mA for protein separation. Gel contents were then transferred to nitrocellulose membranes at 250 mA for 2 h. Free binding sites were blocked in 2% (w/v) bovine serum albumin in 20 mmol/L Tris-HCl buffer (pH 7.5) containing 150 mmol/L NaCl and 0.05% Tween 20 for 1 h. LOX antibody was then applied for 2 h. Horseradish peroxidase-conjugated secondary antibody was applied for 1 h. After extensive washing, protein bands were visualized with Amersham ECL Western Blotting Detection Reagent (Little Chalfont, Buckinghamshire, UK). Membranes were then stripped in the 25 mmol/L glycine-HCl (pH 2.5) buffer containing 2% SDS and reprobed with *β*-actin antibody to confirm equal protein loading.

### 2.11. Statistical Analysis

SPSS 13.0 for Windows (SPSS, Inc., Chicago, IL, USA) was used for analysis. Data are presented as the mean ± standard error (SE). We first analyzed the normality of all the data and showed that the results of TC, HDL, LDL, motor NCV, sensory NCV, mean dendrite length, and VEGF were normally distributed; thus, unpaired Student's *t*-tests were used for statistical comparisons and Pearson's tests were used for correlation analysis. The results of TG, thermal response latency, and intraepidermal nerve fiber density were abnormally distributed; thus, the Mann-Whitney *U* test was used for statistical comparisons and Spearman's rho was used for correlation analysis. Significance was defined as a *P* < 0.05.

## 3. Results

### 3.1. HFD-Fed Mice Manifest IGT and Obesity

An intraperitoneal glucose tolerance test (IPGTT) was performed at a fixed time every other week. Consistent with the results in our pilot study [15], after 14 weeks the AUC of blood glucose was higher in the HFD-fed mice compared with control mice, indicating IGT. After 14 weeks, body weight was higher in the HFD mice compared with control mice (34.31 ± 3.23 versus 26.25 ± 1.26 g, *P* < 0.01).

### 3.2. HFD-Fed Mice Develop Dyslipidemia

**Figure 1:**
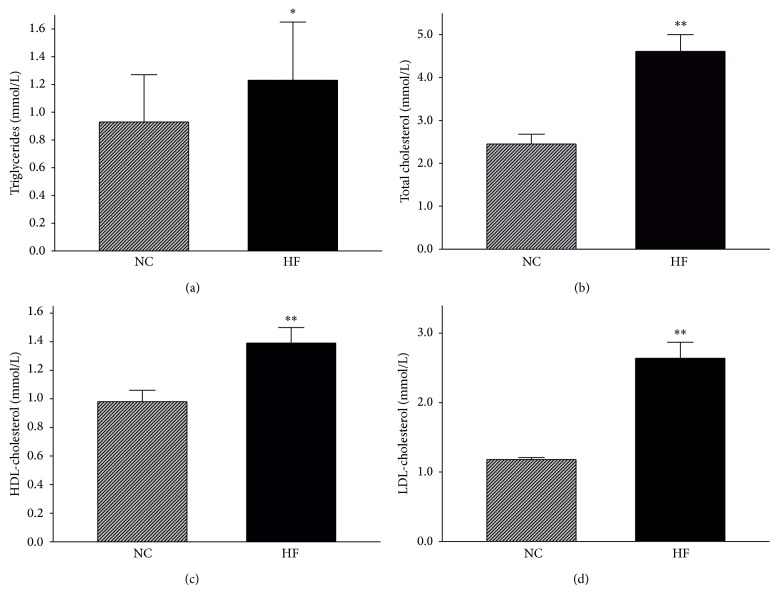
Serum lipids: TG (a), TC (b), HDL-C (c), and LDL-C (d). NC and HF, abbreviations for “normal diet” and “high-fat diet,” represent the control and HFD-fed groups, respectively. Data are presented as the mean ± SE, *n* = 15 mice/group, ^*^ *P* < 0.05, and ^**^ *P* < 0.01.

TG, TC, HDL-C, and LDL-C were increased by 32.3% (*P* < 0.05), 88.16% (*P* < 0.01), 41.84% (*P* < 0.01), and 83.33% (*P* < 0.01), respectively, in HFD-fed mice compared to control mice (Figure 1).

### 3.3. HFD-Fed Mice Had Peripheral Neuropathy

**Figure 2:**
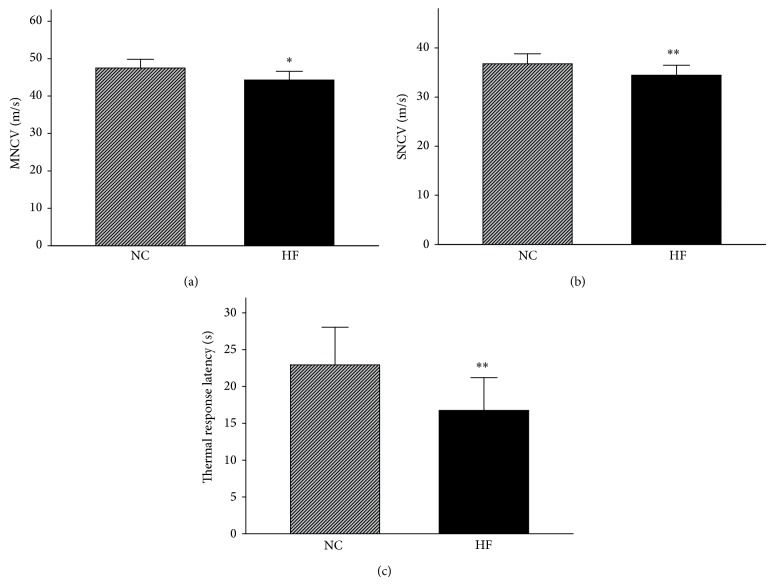
Motor nerve conduction velocity (a), sensory nerve conduction velocity (b), and thermal response latency (c). NC and HF, abbreviations for “normal diet” and “high-fat diet,” represent the control and HFD-fed groups, respectively. Data are presented as the mean ± SE, *n* = 15 mice/group, ^*^ *P* < 0.05, and ^**^ *P* < 0.01.

**Figure 3:**
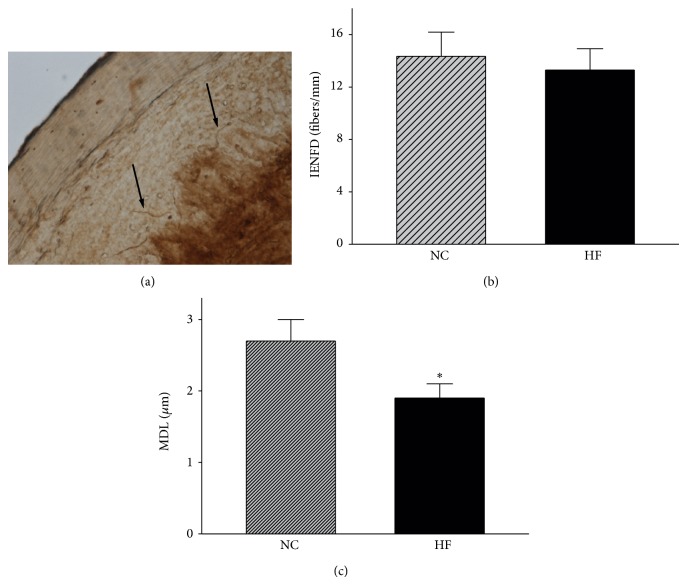
Intraepidermal nerve fiber profiles. (a) Representative image (original magnification ×200). (b) Intraepidermal nerve fiber density. (c) Mean dendrite length. NC and HF, abbreviations for “normal diet” and “high-fat diet,” represent the control and HFD-fed groups, respectively. Data are the mean ± SE, *n* = 15 mice/group, and ^*^ *P* < 0.05.

HFD-fed mice clearly exhibited motor and sensory NCV deficits compared with control mice (*P* < 0.05 and *P* < 0.01, resp.), and the latency of hind-paw withdrawal in response to noxious thermal stimulus was significantly decreased in HFD-fed mice (*P* < 0.01; Figure 2). Intraepidermal nerve fiber densities were similar in the two groups (*P* > 0.05; Figures 3(a) and 3(b)), but the mean dendrite length was significantly decreased in the HFD-fed mice compared with control mice (*P* < 0.05; Figure 3(c)).

### 3.4. Epidermal VEGF-A Immunohistochemistry

**Figure 4:**
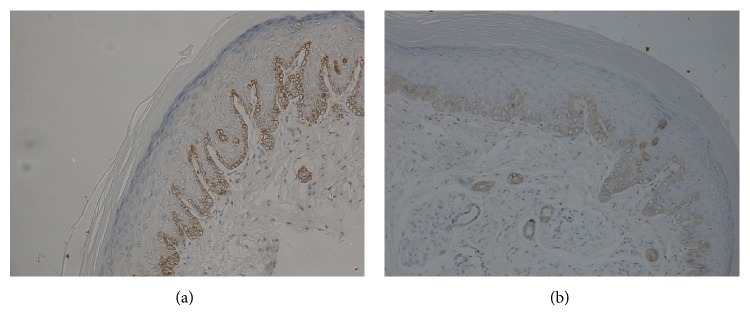
Immunohistochemical analysis of VEGF-A in the epidermis of mice fed a normal (a) or a high-fat diet (b). Original magnification ×400.

The amount of VEGF-A staining was quantified as a percentage of the total area; VEGF-A staining was prominent in the basal and spinous epidermal layers and was significantly reduced in HFD-fed mice (35 ± 4%) compared with control mice (48 ± 6%; *P* < 0.05; Figures 4(a) and 4(b)).

The levels of VEGF are strongly correlated with those of mean dendrite length (*P* < 0.05, *r* = 0.81).

### 3.5. 12/15-LOX Protein Expression

**Figure 5:**
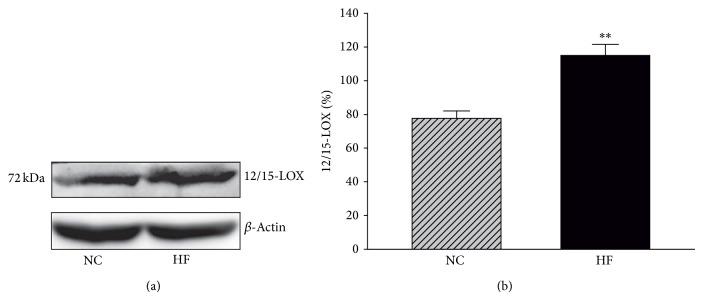
(a) Western blot analyses of sciatic nerve 12/15-LOX. Equal protein loading was confirmed with *β*-actin antibody. (b) Densitometric analysis of 12/15-LOX expression in sciatic nerve. ^**^ *P* < 0.01. NC and HF, abbreviations for “normal diet” and “high-fat diet,” represent the control and HFD-fed groups, respectively.

We determined the expression of 12/15-LOX protein in HFD-fed and control mice. As shown in Figures 5(a) and 5(b), HFD-fed mice had 12/15-LOX upregulation in peripheral nerves. Expression of 12/15-LOX in sciatic nerves increased by 32% in HFD-fed mice compared with control mice (*P* < 0.05).

## 4. Discussion

The HFD-fed C57BL/6 mouse is a commonly used animal model for studying IGT, obesity, and dyslipidemia. Because mice lack cholesterol ester transfer protein, the majority of plasma cholesterol is transported in HDL. Thus, LDL levels are constitutively low [19]. In the present study, HFD-fed mice manifested obesity and IGT, accompanied by significantly increased TC, TG, HDL-C, and LDL-C. In addition, our results confirm previous findings [8, 9] that HFD-fed C57BL/6 mice develop prediabetes and neuropathy, and this may be associated with changes in LOX and VEGF, as indicated by the strong correlations with the mean dendrite length.

In the present study mice fed HFD for 14 weeks resulted in motor and sensory NCV deficits, which is consistent with previous reports [8, 9]. It is known that NCV determines the function of large myelinated nerve fibers, but it is not sensitive enough to assess small nerve fibers and unmyelinated nerve fiber injuries. Early DPN changes mainly occur in small, unmyelinated, and thinly myelinated fibers, which are more sensitive to thermal sensation and present as hypoalgesia or hyperalgesia.

We found that HFD-fed mice displayed significant thermal hyperalgesia, thus implying small sensory nerve fiber lesions [7, 20]. This result is in contrast with reports from others who reported hypoalgesia [8, 9]. Generally, mice with short-term diabetes develop painful neuropathy, whereas mice with longer-term diabetes typically display manifestations of painful and insensate neuropathy or insensate neuropathy alone [21]. In one study [8], mice were fed HFD for 16 weeks, which was longer than the present study, and we speculate that the different phenotypes might be caused by different durations of induction. The mice in the other study [9] had a shorter disease course, which would not account for the discrepant findings. Two similar studies used C57BL/6 mice; one study used a streptozotocin-induced diabetes model for 12 weeks and reported thermal hyperalgesia [22] and the other study described thermal hypoalgesia [23]. It has been suggested [24] that dietary differences might contribute to the dichotomous neuropathic phenotypes observed in C57BL/6 mice.

Peripheral nerve fiber loss has been reported in DPN patients and even in prediabetic patients [7, 16]. Intraepidermal nerve fiber density and mean dendrite length are quantitative indicators that can be used to assess early neuropathy. Previous studies [25] indicated a high sensitivity of intraepidermal nerve fiber density in diagnosing small fiber neuropathy. Indeed, intraepidermal nerve fiber density is significantly reduced in diabetic patients with minimal neuropathy [25] and is related to pain symptoms and neuropathic deficits [26]. Of note, whether or not intraepidermal nerve fiber density loss occurs in prediabetic humans is controversial [6, 7], and there have been no reports of reduction in intraepidermal nerve fiber density in HFD-induced neuropathy in mouse studies [8, 9].

Our study did not show a reduction in intraepidermal nerve fiber density in HFD-fed mice, which is consistent with previously published reports [8, 9]; however, the mean dendrite length was significantly reduced in the plantar skin of HFD-fed mice and appeared to be a more sensitive indicator for early peripheral neuropathy, as reported in clinical trials [7, 16], suggesting that the mechanisms for reducing fiber length and density are probably different. Indeed, the mean dendrite length might directly reflect the pathologic mechanism for fiber reduction.

In the present study VEGF-A expression in the plantar skin of HFD-fed mice was significantly less than controls. This finding indicated that vascular factors might contribute to HFD-induced neuropathy [17, 27]. VEGF was originally identified by its ability to promote vascular endothelial mitosis and new blood vessel formation [17]. VEGF is an angiogenic factor, reflecting the degree of neovascularization in diabetic complications [27]. Furthermore, it has been shown that VEGF has neuroprotective properties [28]. In a clinical study, the intensity of epidermal VEGF-A staining was significantly reduced in patients with DPN compared to nondiabetic control subjects [29]. Furthermore, it has been reported that gene transfer of an engineered transcription factor promoting endogenous VEGF-A expression could prevent experimental DPN [30].

Vascular factors are thought to play a central role in the development of DPN [31]. Neuropathy may develop as a result of endothelial dysfunction, which can cause reduced blood flow. Indeed, lower endoneural capillary density is associated with decreased myelinated fiber density in DPN patients, which may impair their sensory and autonomic neuronal functions. Our finding suggests that neuropathy was associated with similar changes. This is the first evidence suggesting that vascular factors might participate in HFD-induced neuropathy in mice. Also, according to a clinical study [32], impaired skin microvascular reactivity might also play a role in the pathogenesis of pain in DPN. In the present study, HFD-fed mice exhibited thermal hyperalgesia, which might partly be because of vascular changes. Furthermore, our study suggested that the changes of VEGF are correlated with mean dendrite length, a marker of DPN.

Oxidative-nitrosative stress is a well-recognized mechanism in DPN. The role of oxLDL/LOX-1 in dyslipidemia-induced neuropathy has been reported in mice [9]. Our pilot study [15] showed that HFD increased T-SOD activity and MDA content in mice, suggesting an oxidant/antioxidant balance disorder. Recently, it has been reported that 12/15-LOX overexpression and activation might directly contribute to oxidative-nitrosative stress and proinflammatory responses in DPN [33] and attenuating of DPN. Moreover, 12/15-LOX can convert arachidonic acid to 12(S)- and 15(S)-hydroxyeicosatetraenoic (HETE) acids [33], which indirectly induce oxidative stress and proinflammatory responses.

It has been shown that a LOX gene deficiency might prevent HFD by increasing macrophages and monocyte chemoattractant protein-1 overexpression in mouse visceral fat [34], suggesting its role in HFD-induced inflammation. A more recent study [35] demonstrated that 12/15-LOX is implicated in peripheral neuropathy in mice with type 1 and early type 2 diabetes and that HFD-induced early type 2 diabetic mice exhibit LOX upregulation in the sciatic nerve and spinal cord, as well as 12(S)-HETE accumulation. Our finding provides additional evidence for increased LOX activity in the sciatic nerves of prediabetic mice. Furthermore, this finding is also supportive of the recent finding of improved diabetic neuropathy with 12/15-LOX inhibitor [18].

## 5. Conclusion

Our findings confirm that HFD induces neuropathy in C57BL/6 mice and increases tissue oxidative stress prior to the development of overt diabetes. Moreover, the HFD-fed mice in our study displayed thermal hyperalgesia, and this result might provide some insight into why some patients develop painful neuropathy and others develop insensate neuropathy. This is the first study demonstrating that HFD-induced neuropathy is affected by both oxidative stress and vascular factors; intervention with either could be a therapeutic target for DPN. The results also support the notion that mean dendrite length might be a more sensitive marker than intraepidermal nerve fiber density for early neuropathy.
